# Pulmonary Endarterectomy in a Patient with Immune Thrombocytopenic Purpura

**DOI:** 10.1155/2015/898371

**Published:** 2015-05-21

**Authors:** Bedrettin Yıldızeli, Mehmed Yanartaş, Sibel Keskin, Işık Atagündüz, Ece Altınay

**Affiliations:** ^1^Department of Thoracic Surgery, Marmara University School of Medicine, Istanbul, Turkey; ^2^Department of Cardiovascular Surgery, Kartal Koşuyolu Training and Research Hospital, Istanbul, Turkey; ^3^Department of Chest Diseases, Muğla Sıtkı Koçman University, Muğla, Turkey; ^4^Department of Hematology, Marmara University School of Medicine, Istanbul, Turkey; ^5^Department of Anaesthesia, Kartal Koşuyolu Training and Research Hospital, Istanbul, Turkey

## Abstract

Immune thrombocytopenic purpura (ITP) patients are at high risk for bleeding complications regarding surgeries involving cardiopulmonary bypass. We report an ITP patient with chronic thromboembolic pulmonary hypertension who underwent uncomplicated pulmonary endarterectomy with receiving postoperative intravenous immunoglobulin (IVIG) therapy. The positive outcome of this case may suggest that pulmonary endarterectomy surgery is performed safely for ITP patients.

## 1. Introduction

Immune thrombocytopenic purpura (ITP) is a common hematological disorder which involves immune mediated platelet destruction and impaired platelet production [1]. It is characterized by isolated thrombocytopenia in the absence of other causes or disorders that may be associated with thrombocytopenia [1]. The association of ITP and chronic thromboembolic pulmonary hypertension (CTEPH) represents a complex therapeutic challenge. To our knowledge, no case was reported to describe management of patients with ITP undergoing pulmonary endarterectomy (PEA). We describe our experience of perioperative management in a patient of ITP who underwent surgery.

## 2. Case Report

A 69-year-old female with shortness of breath (New York Heart Association Class IV) and CTEPH for a 1-year period was referred for PEA. Her medical history revealed that she had hypertension, regulated with oral antihypertensive drugs, and type II diabetes mellitus. Routine hematological examination was normal with a platelet count of 228,000/mm^3^. Her hematologic investigations showed a provisional diagnosis of ITP for 12 years with history of splenectomy 10 years ago. The patient was on chronic corticosteroid and azathioprine therapy on admission.

**Figure 1:**
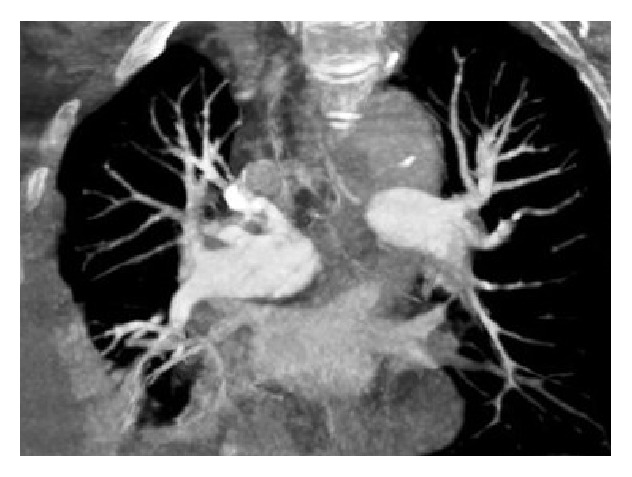
Computed tomogram shows right pleural effusion, web-like filling defects, and endoluminal thromboemboli extending into the right and left pulmonary artery.

**Figure 2:**
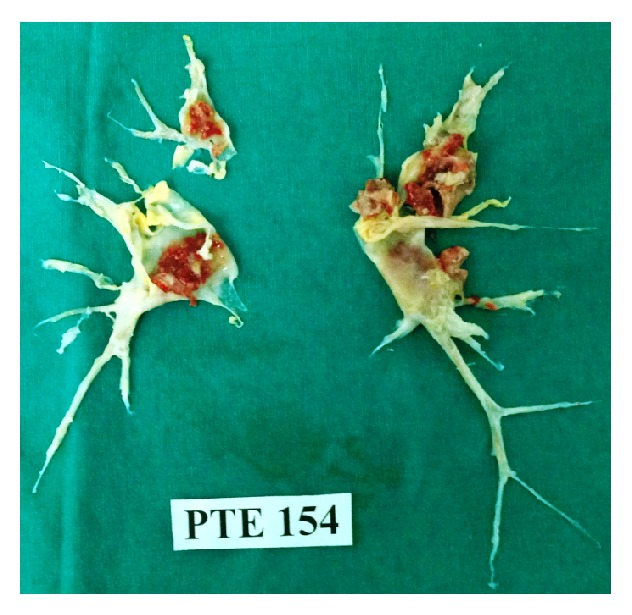
Photograph shows the specimen from our patient's pulmonary endarterectomy.

Her echocardiogram revealed a dilated right ventricle with reduced systolic function and moderate tricuspid regurgitation, but normal left ventricular function with an ejection fraction of 65%. A right heart catheterization revealed slightly elevated pulmonary artery (PA) pressure of 45/16 mm Hg (mean, 28 mm Hg) and cardiac output (CO) of 3.3 L/min. Calculated pulmonary vascular resistance (PVR) was 436 dynes/s/cm^−5^. Spiral computed tomographic scanning of her chest showed right pleural effusion and web-like filling defects and endoluminal thromboemboli in her pulmonary vasculature on the right and left lower lobe sides consistent with chronic thromboembolic disease (Figure 1). Ventilation perfusion scanning confirmed the diagnosis of CTEPH and the patient was scheduled for pulmonary endarterectomy. The PEA surgery was performed as previously described [2]. In brief, surgery was performed under general anaesthesia through a median sternotomy and using extracorporeal circulation. After anticoagulation with heparin, activated clotting time (ACT) of 480 seconds was attained and cardiopulmonary bypass (CPB) was initiated. The endarterectomy specimen was circumferentially followed down to the segmental and subsegmental branches of the pulmonary artery in each lobe, until a complete endarterectomy of the pulmonary vascular bed was achieved with periods of circulatory arrest under deep hypothermia (20°C) for the right and left pulmonary arteries (Figure 2). Patient weaned from CPB without any difficulty and her PVR was decreased to 250 dynes/s/cm^−5^. Bleeding was checked thoroughly and heparin was reversed with protamine to normalize ACT. The chest was closed and the patient was transferred to the intensive care unit and put on ventilator with stable hemodynamics. Six hours later, platelets counts were 158,000/mm^3^. On the first postoperative day (pod), the patient was put on oral azathioprine 50 mg and oral prednisolone 16 mg daily and subcutaneous injections (s.c.) of low-molecular-weight heparin (LMWH) 0.6 cc twice daily. The platelets counts were 241,000/mm^3^. On the 2nd pod platelet counts were decreased to 57,500/mm^3^, and, instead of LMWH, an indirect factor Xa inhibitor fondaparinux was initiated 2.5 mg/day s.c. and dose of prednisolone was increased to 100 mg daily. After three days of treatment, a low platelet count of 57,000/mm^3^ was observed. Steroid therapy was stopped and intravenous immunoglobulin (IVIG) was started at a dose of 400 mg/kg/day for 5 days. Patient was extubated on 5th pod with stable hemodynamics and blood gases. In the meantime the platelet concentrates were transfused between first and 4th pod. Platelet counts were assessed daily and showed a rising trend. On 7th pod, the platelets counts were 183,000/mm^3^. And she was transferred to the ward. IVIG was switched to oral prednisolone treatment, and also azathioprine, fondaparinux, and warfarin therapy was continued. The patient was discharged on 14th pod with a platelet count of 125,000/mm^3^ and with substantial functional and hemodynamic improvement and with advice to continue her medications azathioprine 50 mg and prednisolone 16 mg daily and regularly follow up with a hematologist.

## 3. Discussion

Immune thrombocytopenic purpura is characterized by an abnormally low platelet count of unknown cause. IgG antiplatelet autoantibodies are produced against the platelet glycoprotein IIb/IIIa or GPIb/IX in about 75% of patients causing both platelet destruction and inhibition of thrombopoiesis [1]. The main problem in patients of ITP is an increased risk of bleeding although bleeding symptoms may not always be present. Concomitantly, the ITP patients also present an increased risk of thrombosis related to the presence of hemostatic factors and chronic steroid therapy.

On the other hand, several investigators have recently found in control retrospective and prospective cohort studies that splenectomy is a risk factor of CTEPH [3]. Splenectomy is associated with venous thrombosis in general and, in particular, with nonresolving and recurrent thrombosis and deep vein thrombosis. One late consequence of nonresolution of venous and pulmonary thromboemboli is CTEPH.

Pulmonary endarterectomy is only curative therapy of CTEPH [4]. The technical details of PEA surgery are well established. By using extracorporeal circulation and circulatory arrest under deep hypothermia (20°C), endarterectomy specimen is circumferentially followed down to the segmental and subsegmental branches of the pulmonary artery. Although there is no contraindication for PEA surgery, operability of the CTEPH patient should be determined by an experienced PEA team [4].

Bleeding after CPB surgery is common with about 7% of patients requiring reoperation to control bleeding [5]. Preoperative thrombocytopenia, CPB, deep hypothermia, and induced thrombocytopenia as well as platelet dysfunction and postoperative anticoagulation are expected to increase risk of pericardial effusion and cardiac tamponade. Chowdhry et al. [5] reported a patient with ITP with severe coronary artery disease and mitral regurgitation who underwent CABG and mitral valve replacement. Although early outcome of the surgery was uncomplicated, the patient was dead three weeks later with a diagnosis of pericardial tamponade.

The main treatment of ITP consists of corticosteroids and IVIG. Second and third line therapies, including rituximab, splenectomy, thrombin receptor agonists (TPO-A), and immune-suppressants, are often successful and may cause a long-term increase in the platelet counts [5]. Patients with asymptomatic mild or moderate thrombocytopenia can be followed up with no treatment as platelet counts greater than 5 × 10^4^/mm^3^ are usually not associated with clinically important bleeding. Although platelet transfusion may be short acting, it is useful in instances of severe hemorrhage. Infusion of IVIG has an immediate effect in increasing platelet counts as it blocks the crystallizable fragment (Fc) receptors of macrophages, thus avoiding destruction by phagocytosis.

Jubelirer et al. [6] reported that patients with ITP presenting with mild or moderate thrombocytopenia can be successfully supported to control bleeding during or after coronary artery bypass grafting (CABG) with IVIG and/or platelet transfusions.

The present patient had a diagnosis of ITP and CTEPH disease and underwent successful PEA. To our knowledge, no case of PEA for a patient with a history of ITP has previously been reported. She had an uneventful intraoperative course and there was no difficulty in obtaining surgical hemostasis. In the postoperative period also the patient did not have much bleeding and bleeding related complications. The decrease in the platelet count was managed successfully with platelet transfusion, steroids, and IVIG. Patient was discharged with acceptable platelet counts and with advice about taking anticoagulants, timely INR monitoring, and regular follow-up.

We conclude that PEA surgery can be performed safely to a patient with CTEPH associated ITP. We would recommend that, with an accurate and precise postoperative management, ITP is not contraindicated for PEA surgery.
